# The Young Stethoscopist; or, the Student's Aid to Auscultation

**Published:** 1846-07

**Authors:** 


### ART. XVIII.

*The Young Stethoscopist; or, the Student's Aid to Auscultation.* By H. J. BOWDITCH, M.D.—*Boston, (U. S.)* 1846. 12mo, pp. 248.

THIS is, in one respect, a very original book on auscultation. It includes a description of that method of diagnosis in its applications to the lower animals; it is illustrated by a variety of woodcuts representing square pieces of India-rubber (p. 39), numerous stethoscopes, and a horse (p. 224); while its sections are headed with pictorial letters, after the manner of our facetious friend Punch. All this may not be without its utility, as the book is expressly designed for junior auscultators. Our notice of its contents will be limited to comments on a few points which have struck us in turning over the pages.

Dr. Bowditch appropriates the phraseology and observations of writers on this side the Atlantic, with very imposing coolness; at this we do not feel any surprise: but how does Dr. Bowditch venture to lay hands on the property of his neighbours of Philadelphia (Dr Gerhard is *located* there, we believe,) without some fear of exposure? He gives the exact explanation long since tendered by the Philadelphian physician, of the natural state of prolonged expiration at the apex of the right lung, without the slightest reference to its author's name. We ourselves doubt extremely whether Dr. Gerhard's be the true explanation of the fact; but, true or false, the idea of the phenomenon being dependent on the greater width of the right than the left bronchus, should have been conceded to Dr. Gerhard.*

Dr. Bowditch has twice heard the "pulmonary crumpling sound" (of Fournet) in "apparently early stages of tubercular disease. It was connected with no other physical sign, and occurred at the end of inspiration. Both patients had had hemoptysis; both apparently regained their health."

In certain cases of bronchitis, both acute and chronic, says Dr. Bowditch, "you may get no physical signs." This is true most perfectly, if understood to apply to the ruder class of signs, as rhonchi and great change in the sound of percussion; it is untrue, if referred to trifling alteration in the respiratory murmur.

"Twice" Dr. Bowditch "has observed a very curious phenomenon in the second stage of pneumonia of the posterior portions of a lung; viz.

* Or, if not to him, to Fournet, who taught the same doctrine, and illustrated it by a figure.

an almost tympanitic state of the breast." (p. 54.) Would not any reader infer from this passage that the observation it records is a novel one? yet the learned doctor is doubtless—or at least ought to be—as fully alive as ourselves to the fact, that the occasional occurrence of tympanitic resonance in pneumonia has for years been matter of established knowledge in these countries.

"In certain cases," observes the author, "there is an *augmentation* of the natural sounds on percussion, even when there are many tubercles." This we consider a correct observation, as also the explanation offered that surrounding emphysema is the source of this unusual condition. But, practically speaking, the fact is of little importance from its rarity: it is rare, as we conceive, because when emphysema to any extent is developed round tubercles, these have already increased sufficiently in quantity to deaden the sound, in spite of the coexistent dilatation of the vesicles.

Dr. Bowditch's "method of examining a case" of suspected phthisis, though not exhibiting a particle of novelty, and omitting all investigation of certain delicate points of which the refined auscultator knows the occasional importance, is the best thing in the division of his book referring to the lungs. What he has given, he has given clearly, and if his directions be followed, and the intellectual qualifications of the observer be not particularly obtuse, he will rarely fail in a correct diagnosis. Even this modified praise, however, cannot be extended to the observations on acute phthisis, and phthisis in children: seven lines and a half of peculiarly meagre matter contain the sum of what is said of both.

Talking of the veins, Dr. Bowditch says, that under certain circumstances a "sound called *bruit de diable*, or *devil's noise* [!] is heard in them. It was named by Bouillaud, and resembles the sound produced by a toy. It is similar to the bellows-murmur of the arteries, except that it is continuous." This short statement contains two errors; but these are of little consequence. Our motive for referring to the passage is to comment, in a gentle phrase or two, concerning the progress of knowledge of M. Bouillaud and his countrymen on the subject of venous murmurs. M. Bouillaud baptized the venous murmur of continuous type with irregular increase of force, as above stated—*humming-top noise;* and upon this wonderful achievement has had his praises echoed far and wide for years. But baptizing the thing was not enough—a full description of its characters, origin, relations, significations, source, of what it did do, and what it didn't do—must follow, for M. Bouillaud had a gaping auditory, who had decreed him the office of "high priest of cardiac disease," and he must deliver oracles, whether the spirit moved him or not. And the oracle spake, and spake with a vengeance! It is needless to remind our readers of the error of Bouillaud in maintaining venous murmurs to have their seat in the arteries, or of the thrice absurd inferences into which he fell, through the influence of that primary error. It is enough to state, that so late as 1841 Messrs. Barth and Roger, in the first edition of their 'Manual on Auscultation,' gravely circulated the mass of incorrect statement given forth by the oracle. And in the second edition of their work (1844), something worse than their previous ignorance prevails, in their notice of venous murmurs. "Ward and Hope," say they (p. 498), "admitted that the continuous murmur is produced in the veins." "But

*their theory was not accepted,*" add these writers; a fact new to us, we confess, for never was there a fact in clinical observation, within our memory, so instantly received as a truth as this very one. But M. Bouillaud and his clique of course did not "accept" it, the writers of the 'Manual' mean; and why should they? Dr. Johnson once said of our friends on the other side of the Tweed—"that Scotchman must be a sterling moralist, who would prefer truth to Edinburgh:" our *chers confrères* across the straits of Dover go further—they do not recognize any truth except their own truth. Even *now*, these French writers cannot bear to disguise themselves by being for once just to foreigners. Is it not monstrous, to find them coolly telling us that it is only since one M. Aran (an inhabitant of Paris) examined the subject, that the venous theory is admitted!

A prominence exists sometimes in the precordial region, in cases of pericardial effusion and hypertrophy. But the measure of this prominence? Happily the age we live in has given birth to a certain Dr. Felix Andry, in whom the *mens divinior* exhibits itself in the invention of a "cystometer," —only think, a *cystometer*,—to take an exact measurement of the same; henceforth, no man attending a case of pericarditis need pine in hopeless anxiety as to the number of hairs' breadth, more or less, the precordial surface may have risen in his suffering patient!

But to return to Dr. Bowditch. We do not admire books superficial and imperfect, whether these characters be aimed at by their authors or not: we therefore do not admire this book. Secondly, we do not admire bad grammar or bad English; and as we find numerous specimens of both, —*e. g.* in pages 6, 9, 55, 157,—we do not admire this book. And, lastly, we do not admire books which tell the world nothing more than many of similar title: now as this book does not tell a sixth as much as, and that sixth not better than, its predecessors, we neither admire it, nor see any necessity for its production.

